# Direct modulation of microglial function by electrical field

**DOI:** 10.3389/fcell.2022.980775

**Published:** 2022-09-08

**Authors:** Anton Lennikov, Menglu Yang, Karen Chang, Li Pan, Madhu Sudhana Saddala, Cherin Lee, Ajay Ashok, Kin-Sang Cho, Tor Paaske Utheim, Dong Feng Chen

**Affiliations:** ^1^ Department of Ophthalmology, Harvard Medical School, Schepens Eye Research Institute of Massachusetts Eye and Ear, Boston, MA, United States; ^2^ Department of Medical Biochemistry, Oslo University Hospital, University of Oslo, Oslo, Norway; ^3^ School of Optometry, The Hong Kong Polytechnic University, Hong Kong, Hong Kong SAR, China; ^4^ Wilmer Bioinformatics, Johns Hopkins University School of Medicine, Baltimore, MD, United States; ^5^ Department of Ophthalmology, Oslo University Hospital, University of Oslo, Oslo, Norway

**Keywords:** electric stimulation, microglia, bulk RNA sequencing, cell motility, inflammation, phagocytosis, BV-2

## Abstract

Non-invasive electric stimulation (ES) employing a low-intensity electric current presents a potential therapeutic modality that can be applied for treating retinal and brain neurodegenerative disorders. As neurons are known to respond directly to ES, the effects of ES on glia cells are poorly studied. A key question is if ES directly mediates microglial function or modulates their activity merely via neuron-glial signaling. Here, we demonstrated the direct effects of ES on microglia in the BV-2 cells—an immortalized murine microglial cell line. The low current ES in a biphasic ramp waveform, but not that of rectangular or sine waveforms, significantly suppressed the motility and migration of BV-2 microglia in culture without causing cytotoxicity. This was associated with diminished cytoskeleton reorganization and microvilli formation in BV-2 cultures, as demonstrated by immunostaining of cytoskeletal proteins, F-actin and β-tubulin, and scanning electron microscopy. Moreover, ES of a ramp waveform reduced microglial phagocytosis of fluorescent zymosan particles and suppressed lipopolysaccharide (LPS)-induced pro-inflammatory cytokine expression in BV-2 cells as shown by Proteome Profiler Mouse Cytokine Array. The results of quantitative PCR and immunostaining for cyclooxygenase-2, Interleukin 6, and Tumor Necrosis Factor-α corroborated the direct suppression of LPS-induced microglial responses by a ramp ES. Transcriptome profiling further demonstrated that ramp ES effectively suppressed nearly half of the LPS-induced genes, primarily relating to cellular motility, energy metabolism, and calcium signaling. Our results reveal a direct modulatory effect of ES on previously thought electrically “non-responsive” microglia and suggest a new avenue of employing ES for anti-inflammatory therapy.

## Introduction

Non-invasive electric stimulation (ES) is emerging as a therapeutic modality for eye and brain diseases (Sanie-Jahromi et al., 2021). Clinical studies suggested that ES improves retinal function in eye diseases, such as retinitis pigmentosa (Schatz et al., 2017), age-related macular degeneration (AMD) (Shinoda et al., 2008), and central or branch retinal arterial occlusion (Naycheva et al., 2013). The beneficial effects of ES have been mainly attributed to their direct modulatory effects on neurons. While glial cells play critical roles in the pathogenesis of retinal degenerative diseases and undergo morphological and functional changes in responding to electrical activities, the effects of ES on glial cells are poorly understood. Preclinical studies in Gerbil indicated that ES reduced microglia activation and density and decreased the expression of Interleukin 6 (IL-6) and COX-2 in the acute ocular hypertensive injury model (Fu et al., 2018). Currently, it remains unknown if ES can directly act on the microglia or merely modulate their activity via neuron-glial signaling.

Microglia represents a specialized population of macrophage-like cells that form the first line of defense in the central nervous system (CNS), including the retina (Bachiller et al., 2018). They become activated by stressor stimuli, such as infection, ischemia, and trauma, to induce a pro-inflammatory response (Harry and Kraft, 2008). Activated microglia migrate toward the injury and change their ramified morphology to ameboid-like with reduced cellular processes (Rojas et al., 2014). Importantly, they engage in active phagocytosis of cellular debris and bacterial particles and facilitate inflammatory responses by expressing cytokines and neurotoxic factors, such as Tumor Necrosis Factor-α (TNF-α), Interleukin 6 (IL-6), and cyclooxygenase-2 (COX-2) (Lucin and Wyss-Coray, 2009). These actions open the blood-retinal barrier, allowing the recruitment of circulating immune cells to defend the retina or brain (Rashid et al., 2019). However, uncontrolled microglial activation contributes to chronic neurodegeneration in glaucoma and Alzheimer’s disease (Ramirez et al., 2017). Reducing microglial activation and migration capacity is a promising strategy for preserving visual and CNS functions in neurodegenerative disorders.

Due to microglia’s critical and multifaceted roles in retinal and brain neurodegeneration, the direct regulation of ES on their gene expression and function warrants careful examination. While the biological effects of ES may vary depending on the stimulation paradigms, including waveforms, amplitudes, and frequencies/pulse width of the microcurrents (Mohan et al., 2020), our previous studies using low-intensity microcurrent ES have reported prominent responses of Müller glia to the ramp waveform of ES *in vitro* (Enayati et al., 2020). In this study, we explored ES’s cellular and molecular effects on microglia in the presence and absence of a potent inflammatory stimulus, lipopolysaccharide (LPS).

## Materials and methods

### Cell culture conditions

The mouse microglial cell line BV-2 (EOC 20; CRL-2469; Lot 70005904; American Type Culture Collection, ATCC, Manassas, VA, Unites States), derived from the C3H/HeJ female mouse, were used between passages 3–8. BV-2 cells were maintained in HyClone Dulbecco’s Modified Eagle Medium (DMEM)/F12 1:1 (Cytiva life sciences, Marlborough, MA, Unites States). Culture media were supplemented with 10% fetal bovine serum (FBS, Thermo Fisher Scientific, Waltham, MA, Unites States), 1% penicillin/streptomycin (Pen/Strep, Thermo Fisher Scientific, Waltham, MA, Unites States), and 50 ng/ml of recombinant mouse CSF-1 (Peprotech, 315–02, Rocky Hill, NJ, Unites States). In the experiments involving LPS treatment following electric stimulation, BV-2 cells were incubated in the presence or absence of LPS (Sigma Aldrich, St. Louis, MO, US) at 1 μg/ml for 24 h.

### Electric stimulation

The electric stimulation in cultures was performed with STG4000 (Multichannel Systems, Reutlingen, Germany) pulse generator using: a biphasic ramp waveform (100 µAmp, 20 Hz, 1 h); biphasic rectangular waveform (100 µAmp, pulse duration 25 ms, 20 Hz, 1 h) and biphasic sine (100 µAmp, 20 Hz, 1 h). The electric current was delivered to cultures using a c-dish carbon electrode plate (Ion Optix, Westwood, MA, Unites States). Between uses, the c-plate was incubated in 70% ethanol (15 min), followed by a washed with distilled water (15 min), and dried (1 h).

### Imaging

Fluorescent images were obtained with a Leica DMi8 fluorescent microscope and Leica SP8 laser confocal microscope (Leica AG, Wetzlar, Germany).

### Migration assay

BV-2 cells were grown in a 6-well-plate, and the migration assay was conducted in semi-confluent BV-2 cell cultures. A scratch along the diameter of the well was introduced with the 1 ml pipet tip with the gap distance at approximately 1 mm. Then, cultures were electrically stimulated with biphasic ES of a rectangular waveform, ramp waveform, or Sine waveform at 100 μA, 20 Hz for 1 h. Cells were visualized by staining with Calcein AM 1 μg/ml (C3100MP; Thermo Fisher Scientific, Waltham, MA), and the distance between the cell growth fronts and scratch surface area was measured at 0, 24, and 48 h and quantified by the “masked” observer using ImageJ software (https://imagej.nih.gov/ij National Institute of Health, Bethesda, MD, Unites States).

### Immunofluorescence staining

BV-2 cells were fixed in 2% paraformaldehyde (VVR Life Science) for 5 min, permeabilized by incubation in 0.05% Triton X-100 for 10 min, and blocked with 2.5% normal donkey serum for 1 h at room temperature (RT). The samples were then incubated with primary antibodies TNFa (1:100; AB1793, Abcam, Cambridge, MA, Unites States), COX-2 (1:100; MA5-14568, Thermo Fisher Scientific, Waltham, MA, Unites States), β-Tubulin (1:200; MA5-16308, Thermo Fisher Scientific, Waltham, MA, Unites States), or Alexa Fluor™ 647 Phalloidin conjugate (1:100; A22287, Thermo Fisher Scientific, Waltham, MA, Unites States) for 1 h at room temperature and washed with PBS-Tween 20 (0.05%; PBS-T). After PBS-T washing, they were then visualized by goat anti-rabbit IgG (H + L), Alexa Fluor 488 (A-11034, 1:1000, Fisher Scientific, Waltham, MA, Unites States), donkey anti-mouse IgG (H + L), Cyanine3 (A10521, 1:1000; Fisher Scientific, Waltham, MA, Unites States)/ The cell nuclei were visualized by incubation with 4′,6-diamidino-2-phenylindole (DAPI); (1:5000; Sigma). The slides were mounted with a ProLong Diamond antifade reagent (Thermo Fisher Scientific, Waltham, MA, Unites States).

### Scanning electron microscopy (SEM)

The BV-2 cells were cultured on cell-culture-treated coverslips to ensure adhesion. The cells were electrostimulated (Ramp, 100 μA, 20 Hz, 1 h) and were placed in 1/4 Karnovsky’s fixative (1% paraformaldehyde +1.25% glutaraldehyde in 0.1M sodium cacodylate buffer, pH 7.4) for 2 h at room temperature. After fixation, samples were rinsed with 0.1M sodium cacodylate buffer and post-fixed with 1% osmium tetroxide in 0.1M sodium cacodylate buffer for 1 hour. The samples were rinsed in 0.1M sodium cacodylate buffer and distilled water, then dehydrated with graded ethyl alcohol solutions and dried in a Tousimis samdri-795 critical point dryer (Tousimis Research Corporation, MD, USA.) The dried samples were mounted onto an aluminum stub using adhesive carbon tape and sputter-coated with gold using a JEOL DII-29010SCTR Smartcoater (JEOL Unites States Inc., Peabody, MA, USA.) Samples were SEM imaged using a JEOL JCM-7000 Neoscope scanning electron microscope (JEOL Unites States Inc., Peabody, MA, Unites States) at 15 kV with secondary electron image detection for digital TIFF file image acquisition at various magnifications.

### Cell death evaluation

BV-2 cells were cultured in 24-well plates until they reached 80% confluence. The cells were stimulated by biphasic ramp electric currents at 100 μA–1,000 μA, 20 Hz for 1 h. After 24 h of incubation, 100 µL of culture media were collected and added to a lactate dehydrogenase (LDH) assay (Thermo-Fisher Scientific). The media collected from non-stimulated cultures were used as the negative controls, and the media without cells was used as background controls. BV-2 cultures treated with 0.05% Triton-X100 for 30 min were used as a positive control of maximum LDH release. The staining was carried out according to the manufactural instruction, and the resulting absorbances were read at 490 nm using an 800 TS Absorbance Reader (BioTek Instruments, Winooski, VT, Unites States). Absorbances at 650 nm were used as a reference wavelength. For fluorescent detection of cell death, active components of ReadyProbes Cell Viability Imaging Kit (Blue/Green) (R37609, Invitrogen, Thermo Fisher Scientific) 30 μL/ml were added to the culture media and incubated for 30 min producing blue (live) and green (dead) fluorescence. A “masked” observer quantified the number of dead cells in the resulting images.

### Proteome profiler mouse cytokine array

The Proteome Profiler Mouse Cytokine Array Kit (ARY006, Panel A; R&D Systems, Minneapolis, MN, Unites States) was used to detect the presence of pro-inflammatory cytokines in the supernatants collected from BV-2 cultures stimulated with the electric field at 24 h incubation in the presence or absence of and LPS (Sigma Aldrich, St. Louis, MO, US) 1 μg/ml for 24 h. Assays were carried out according to the manufacturer’s protocol as previously described (Chondrou et al., 2020). Briefly, the array membranes were blocked with array buffer for 1 h at the rocking platform. The BV-2 culture supernatants were collected centrifuged at 17,000 g for 10 min at 4C and passed through a 22 µm membrane filter; 1 ml of each supernatant sample was mixed with 0.5 ml of array buffer and incubated with 15 μL of reconstituted mouse cytokine array panel A detection antibody cocktail for 1 h. Following incubation, the samples were added to the blocked array membranes overnight. The array membranes were washed with 1x array washing buffer, incubated with streptavidin-HRP for 30 min, and imaged with a gel detection system iBright (Thermo Fisher, Unites States). Results were analyzed using ImageJ software. The density values were normalized to the LPS treated group.

### Quantitative RT-PCR (qPCR)

The cells were washed with PBS, and total RNA was extracted using Quick RNA Micro Prep Kit 11–328M (Zymo Research, Irvine, CA, Unites States). The RNA was analyzed for quality and was quantified using a NanoDrop ND-1000 (Thermo Fisher Scientific, Waltham, MA) and reverse-transcribed to complementary DNA (cDNA) using Takara Prime Script RT Master Mix RR036A (Takara, San Jose, CA, Unites States) in Applied Biophysics 2,720 thermal cycler (Life Technologies, Waltham, MA, Unites States). Gene expression was analyzed using Power SYBR Green Master Mix (Thermo Fisher Scientific, Waltham, MA, Unites States) using qPCR. The primers used were listed in Table 1; GAPDH or β-actin was used as a housekeeping gene. Reactions were detected using Step One Plus real-time PCR (RT-PCR) system (Applied Biosystems, Foster City, CA, Unites States). The relative expression values of the target genes were normalized to the housekeeping gene, and the fold change was calculated using the relative quantification (2−ΔΔCT) method. Three biological replicates per treatment group were run with three technical replicates.

**TABLE 1:** List of mouse-specific primers used in the study.

Gene	Direction	Sequence
TNFα	Forward	CTG​GGA​CAG​TGA​CCT​GGA​CT
	Reverse	GCA​CCT​CAG​GGA​AGA​GTC​TG
IL-6	Forward	AAG​TGC​ATC​ATC​GTT​GTT​CAT​ACA
	Reverse	GAG​GAT​ACC​ACT​CCC​AAC​AGA​CC
COX-2	Forward	GCG​AGC​TAA​GAG​CTT​CAG​GA
	Reverse	CAG​ACG​CCA​CTG​TCG​CTT​T
Tbkbp1	Forward	AGG​AGC​AAC​TCC​AGG​CGG​AAT​G
	Reverse	AGC​CAT​GTC​ACA​TTC​CGA​CTG​G
Atp2b4	Forward	CAC​CAT​CTC​ACT​AGC​CTA​CTC​TG
	Reverse	AGT​GTG​CCT​GTC​TTA​TCG​GAG​C
GAPDH	Forward	ACT​CCA​CTC​ACG​GCA​AAT​TC
	Reverse	TCT​CCA​TGG​TGG​TGA​AGA​CA
β-actin	Forward	CAT​TGC​TGA​CAG​GAT​GCA​GAA​GG
	Reverse	TGC​TGG​AAG​GTG​GAC​AGT​GAG​G

### Zymosan bioparticles phagocytosis assay

BV-2 cells were electrostimulated with biphasic ramp, rectangular or sine ES (100 μA, 20 Hz, 1 h). Following stimulation, phagocytosis Assay Kit (Red Zymosan) cy3 zymosan bioparticles conjugate (ab234054; Abcam) was added to the culture media at 1 μg/ml. Following 12 h of incubation, BV-2 cells were washed with PBS 3 times and counterstained vitaly with Calcein AM 1 μg/ml (C3100MP; Thermo Fisher Scientific, Waltham, MA) and imaged. A “masked” observer quantified the number of absorbed particles. Non-stimulated cells treated with zymosan bioparticles were used as controls.

### Bulk RNA sequencing and data analysis

RNA samples were isolated as described above, and bulk RNA sequencing was carried out by Novogene Leading Edge Genomic Services & Solutions (California, Unites States). Briefly, the RNA quality was determined using the Agilent bioanalyzer 2,100 (Agilent Technologies, Santa Clara, CA, Unites States). The analysis showed clear, defined 28 and 18s rRNA peaks, indicating high-quality RNA. The volume, concentration, and RIN values of the RNA samples are presented in Supplementary Table S1. RIN value ≥8 was set as the cutoff for sample inclusion for downstream processing for RNA sequencing analysis. RNA samples were submitted to Novogene Leading Edge Genomic Services & Solutions (California, Unites States) for sample preparation and sequencing. The samples were first DNase-treated and assessed for total RNA quality using the Agilent 2,100 Bioanalyzer, followed by two rounds of polyadenylate positive (poly A+) selection and conversion to cDNA. RNA sequencing was performed on the Illumina HiSeq 2,500 (San Diego, CA, Unites States), providing up to 300 GB of sequence information per flow cell. TruSeq library generation kits were used according to the manufacturer’s instructions (Illumina). Library construction consisted of random fragmentation of the poly A+ mRNA and cDNA production using random primers. During the sequencing run, the cDNA ends were repaired, A-tailed, and adaptors ligated for indexing (up to 12 barcodes per lane). Before cluster generation, the cDNA libraries were quantitated using qPCR in a Roche LightCycler 480 with the Kapa Biosystems kit for library quantitation (Kapa Biosystems). The raw fastq reads were trimmed for adapters and pre-processed to remove low-quality reads using Trimmomatic v. 0.36 (Bolger et al., 2014) with default parameters. After trimming, each paired-end sequence file’s quality was evaluated using FastQC (http://www.bioinformatics.babraham.ac.uk/projects/fastqc/) tool. The processed datasets were mapped to the mouse reference genome (mm10) with HISAT2 (http://daehwankimlab.github.io/hisat2/) with default parameters. Finally, both control, LPS, and Ramp + LPS treated comparison transcript counts (matrix file) were used for differential gene expression using the DESeq2 v 1.20 package of R software v 4.0.5 (http://www.R-project.org) with primary parameters such as FDR (false discovery rate), logFC (log fold-change), logCPM (log counts per million), and *p*-value. Unigenes with adjusted *p*-values of less than 0.05 (*p* < 0.05) and the logFC >1.3 were considered significantly differentially expressed genes. The resulting list of genes *p*-value, log10 *p*-value, and log2 FC are presented in supplementary Exel files DEG_LPSvsCtrl and DEG_ES_LPSvsLPS.

### Gene annotation

Gene ontology (GO) Enrichment Analysis (https://david.ncifcrf.gov/) was used for functional annotation and pathways analysis. An adjusted EASE (Expression Analysis Systemic Explore Score) score of 0.05 and threshold count of >2 genes were employed. Benjamin–Hochberg multiple testing correction was applied to the *p*-values. GO terms with FDR q < 0.05 were considered significantly enriched within the gene set (Huang et al., 2007; Saddala et al., 2018). The visualization of gene ontology through dot-plots charts was performed using ggplot2 - Tidyverse software (https://ggplot2.tidyverse.org/).

### Statistical analysis

All experiments were performed in triplicate. Experimental values were expressed as the mean ± SD for the respective groups. Statistical analyses were performed with GraphPad Prism software (https://www.graphpad.com/scientific-software/prism/). The student t-test was used to compare the two groups, and one-way analysis of variance (ANOVA) with Tukey multiple comparisons was used whenever comparing multiple groups. The following designations for the *p*-value were used in the manuscript figures: *p < 0.05; **p < 0.01; ***p < 0.001. Statistical analyses for the RNA seq data were performed using the Trinity software (https://github.com/trinityrnaseq/trinityrnaseq/wiki). Student t-tests and Benjamin–Hochberg corrections (FDR) were also used in the analyses. A *p*-value of less than 0.05 was considered statistically significant.

## Results

### Electric stimulation inhibits microglial migration, microvilli protrusion, and cytoskeleton protein reorganization

**FIGURE 1:**
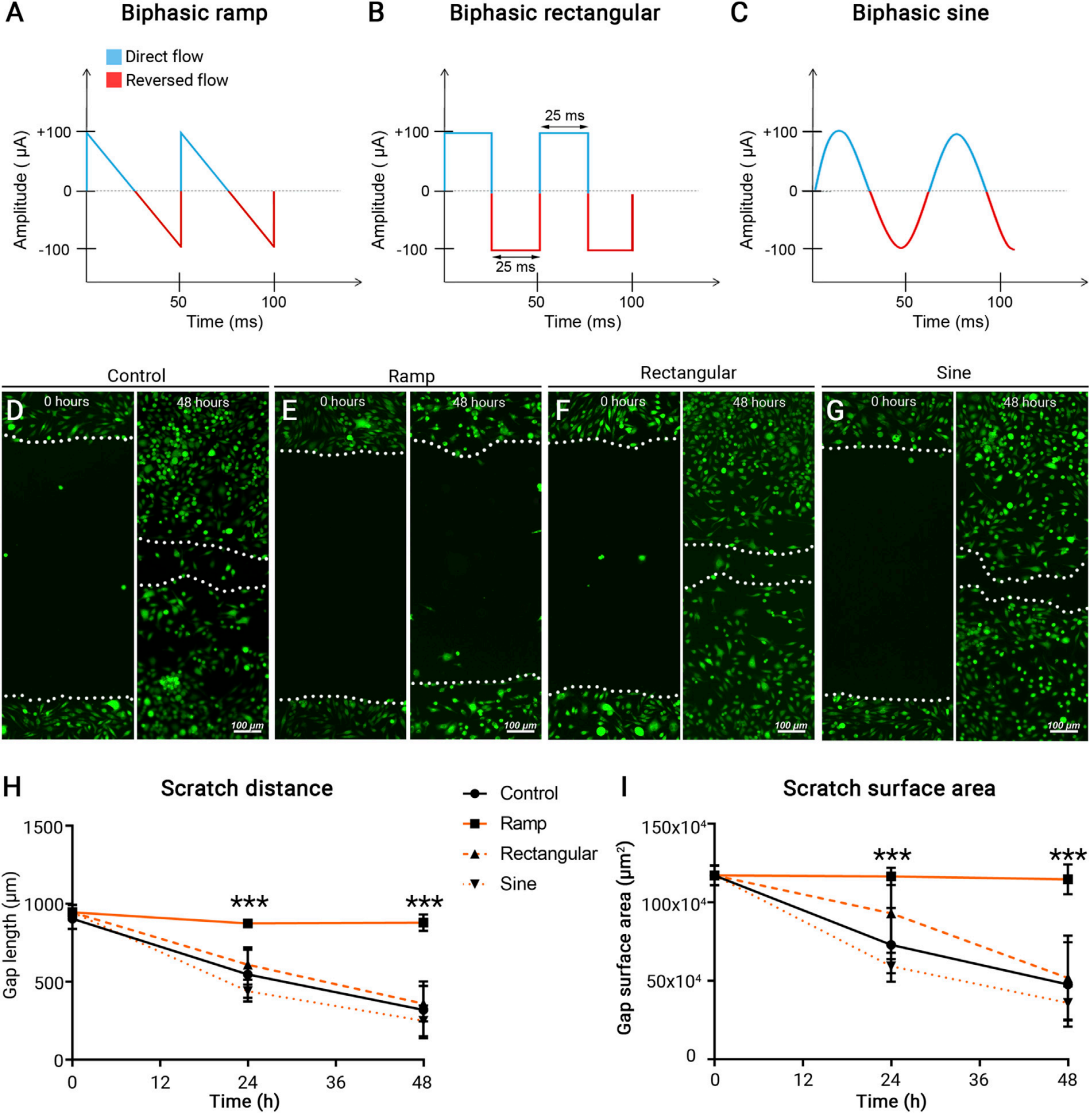
Microcurrent ES of ramp waveform inhibits BV-2 cell migration. **(A–C)** Schematic representation of different ES waveforms: **(A)** Ramp; **(B)** Rectangular; **(C)** Sine. **(D–G)** Photomicrographs of migratory BV-2 cells stained by a fluorescence vital dye Calcein AM (green) in the scratch assay and assessed at 0 and 48 h post scratching. Cells were cultured under a control condition **(D)** or subjected to ES of ramp **(E)**, rectangular **(F)**, or sine **(G)** waveform. Scale bar: 100 µm. **(H,I)** Quantitative analysis of scratch distance **(H)** and scratch surface area **(I)**. *n* = 8 cultures/group; Statistical significance was determined using one-way ANOVA with Tukey multiple comparisons. *p < 0.05; **p < 0.01; value = means ± S.D.

Microglia motility is a strong indicator of its function, particularly in response to pro-inflammatory stimuli (Hikage et al., 2021). To assess the direct impact of the electrical field on microglial function, we investigated the cell motility following ES in cultured BV-2 cells, a murine microglial cell line, using the scratch assay. Our previous studies suggest that while neurons and glial cells respond robustly to electrical microcurrents (100 μA; 20 Hz) (Enayati et al., 2020; Yu et al., 2020), the ramp waveform evokes the most prominent result in glial cells compared to the other waveforms. We compared BV-2’s responses to three different ES waveforms ramp (Figure 1A), rectangular (Figure 1B), and sine (Figure 1C). A ∼1 mm scratch was performed in the confluent BV-2 cultures, and cells were subjected to ES (100 μA; 20 Hz) in one of the three waveforms as indicated. Repopulation of the scratch area was observed 24 and 48 h later. Control BV-2 cells cultured in the absence of ES exhibited rapid closure of the scratch distance and disappearance of the surface area within 48 h after scratch (Figures 1D,H,I). Intriguingly, treatment of BV-2 cells with ES of the ramp waveform, but not rectangular or sine waveforms, resulted in the failure of scratch closure measured at both 24 (*p* < 0.001) and 48 (*p* < 0.001) hours post-ES, when compared with the control group (Figures 1D–I). The results indicate a negative impact of ES of a ramp waveform on microglia motility.

**FIGURE 2:**
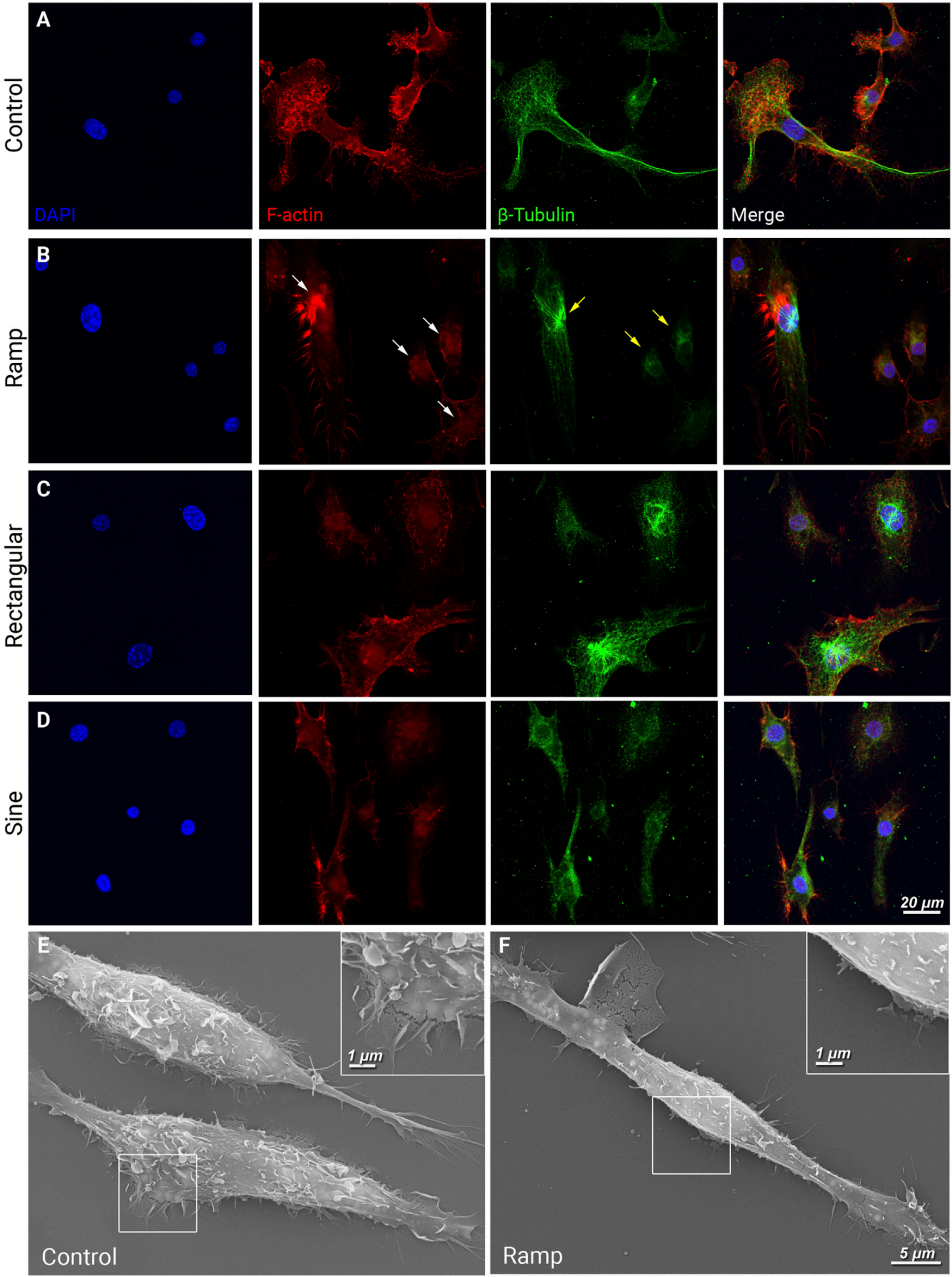
Ramp ES inhibits the redistribution of cytoskeletal and motility proteins and the formation of cellular lamellipodia and microvilli. **(A–D)** Immunostaining of F-actin (red) and β-tubulin (green) in BV-2 cells cultured under a control condition **(A)** after ES biphasic ramp; white arrow indicates redistribution of F-actin within the cell. **(B)**, ES biphasic rectangular **(C)**, or ES biphasic sine **(D)**. Scale bar: 20 µm. **(E,F)** Scanning electron microscopy images of BV-2 cells in control **(E)** and after ES biphasic ramp **(F)**. Inserts reveal cell membrane morphology in the leading edge and lamellipodia. Scale bar: 5 μm; insert: 1 µm.

**FIGURE 3:**
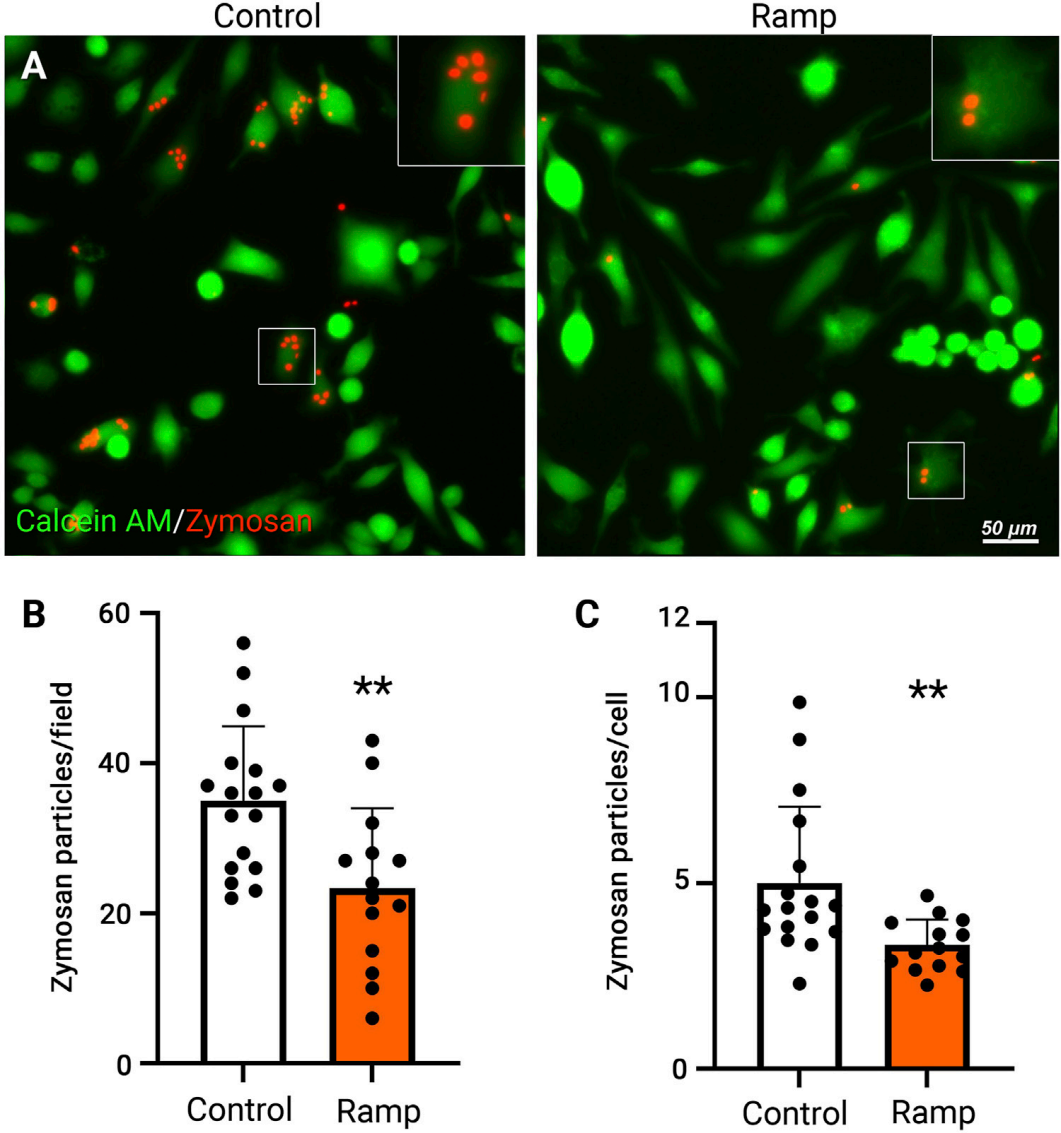
Ramp ES reduces phagocytosis of BV-2 cells. Representative fluorescent images of BV-2 cells cultured under a control condition or ES biphasic ramp **(A)** and incubated with fluorescent (cy3) zymosan particles (red) for 24 h and Calcein AM stain (green) was used to visualize BV-2 cells. Scale bar: 50 µm. Inserts present individual cells with zymosan particles. Quantification of zymosan particles in BV-2 cells in control and ramp ES-treated cultures per field **(B)** and the average number of zymosan particles per cell **(C)**; Control *n* = 17; Ramp *n* = 14. Statistical significance was determined by Student’s t-test. **p < 0.01; value = means ± S.D.

Dynamic reorganization and rearrangement of actin filaments and microtubules underlie cytoskeletal architectural changes and cell migration (Franco-Bocanegra et al., 2019). Immunocytochemistry of F-actin and β-tubulin in control BV-2 cells revealed cytoskeletal structures extending into the well-defined cellular processes and lamellipodia (Figure 2A). BV-2 cells received ES of ramp waveform showed diminished presence and redistribution of F-actin (white arrows) and β-tubulin (yellow arrows) with predominant localization to the perinuclear area (Figure 3B). In contrast, BV-2 cells that received rectangular or sine waveform of ES exhibited similar F-actin and β–tubulin distribution as the control cells, with some changes observed following rectangular ES (Figures 2B–D). Scanning electron microscopy further demonstrated the formation of lamellipodia and microvilli in control BV-2 cells (Figure 2E), which were nearly absent in ramp ES treated BV-2 cells (Figure 2F). Thus, ES with a ramp waveform, but not rectangular and sine, prominently affects F-actin and β-tubulin distribution and organization, reducing the formation of cellular microvilli.

To exclude that ES of ramp waveform induces microglia cytotoxicity, which prevents cell migration and cytoskeleton reorganization, we assessed BV-2 cell survival under various current amplitudes of ramp ES. The cultures of BV-2 cells were treated with ramp microcurrents ranging from 100—1,000 μA. Cell survival was evaluated by measuring Lactate Dehydrogenase (LDH) release 12 h after stimulation (Suppplementary Figure S1). The results indicated no significant increase in cell death at 100 and 250 μA ES compared to control cultures until ES microcurrent reached 500 and 1,000 µA. The results were confirmed using a fluorescent live/death staining kit (Supplementary Figures S1B, C), in that no significant cell death was observed until the microcurrent reached 500 μA or above. Together, these data suggest that ES with a biphasic ramp waveform, at least when at 100 μA, inhibits the migration and cytoskeleton reorganization of microglia without affecting cell survival.

### Electric stimulation decreases microglial phagocytosis

Microglial phagocytosis is associated with inflammatory responses of microglia and neuron or photoreceptors loss under chronic neurodegenerative conditions, such as retinitis pigmentosa (Zhao et al., 2015). Phagocytosis requires locally coordinated complex cytoskeletal rearrangements (Kuiper et al., 2008); we, therefore, evaluated the phagocytotic activities of BV-2 cells with or without ES treatment by incubating the cultures with fluorescent zymosan particles. Quantification at 24 h post-ES revealed a significant reduction in the total number of fluorescent zymosan particles and zymosan particles per cell (Figure 3C, *p* < 0.01) in ramp ES-treated BV-2 cells compared to control cultures (Figure 3). Consistent with our observations of cell motility and cytoskeletal structural changes, rectangular (Supplementary Figure S2C) and sine (Supplementary Figure S2D) waveforms did not significantly (*p* > 0.05) reduce the number of absorbed fluorescent zymosan particles. However, a tendency of reduction was observed in cultures treated by a rectangular waveform stimulation (*p* = 0.093). Thus, ES-treatment with ramp waveform directly inhibits the phagocytotic activities of microglia. From hereon, only ES with a ramp waveform was used and analyzed.

### Electric stimulation attenuates LPS-induced production of pro-inflammatory cytokines by activated microglia

**FIGURE 4:**
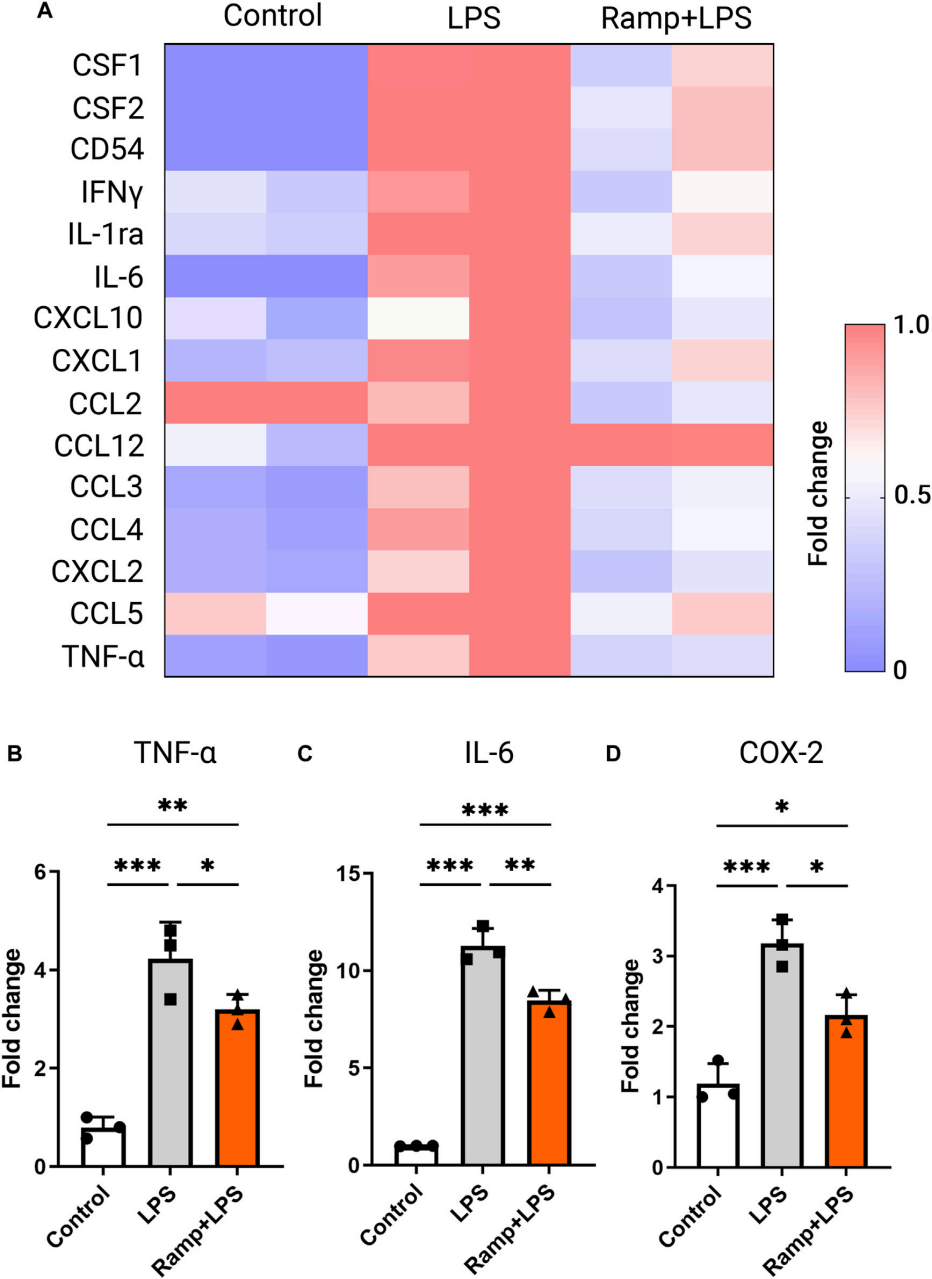
Electric stimulation with ramp waveform reduces BV-2 cell activation in response to LPS stimulation. **(A)** Heatmap of inflammatory cytokine levels as measured by values of densitometry of cytokine arrays and presented as fold changes normalized to the value of the LPS-stimulated group without the ES. RT-PCR **(B–D)** demonstrated changes in levels of expression of TNF-α **(B)**, IL-6 **(C)**, and COX-2 **(D)** in BV-2 cells subjected to ES biphasic ramp at 24 h after LPS stimulation. *n* = 3; Statistical significance was determined using one-way ANOVA with Tukey multiple comparisons. *p < 0.05; **p < 0.01; value = means ± S.D.

Microglia motility and phagocytotic capacity are important aspects of their pro-inflammatory activation. Microglial activation produces pro-inflammatory cytokines such as IL-6 and TNF-α that can be neurotoxic and contribute to the development and progression of neurodegenerative conditions in the brain and retina (Rashid et al., 2019). We next studied the effects of ES on LPS-induced pro-inflammatory cytokine release in activated microglia using a proteome profiler mouse cytokine array. The BV-2 cells were stimulated with ramp ES, followed by incubation with LPS (1 μg/ml), a widely recognized potent activator of microglia (Lively and Schlichter, 2018). As expected, LPS induced the expression of pro-inflammatory cytokines, including Macrophage colony-stimulating factor 1 (CSF1), Macrophage colony-stimulating factor 2 (CSF2), IL-6, IFNγ, TNFα, and C-C Motif Chemokine ligands (CCL) 2, 3, four and 5. ES treatment attenuated the production of all pro-inflammatory cytokines induced by LPS (Figure 4A). ES attenuation of LPS-induced microglial activation was further validated by immunolabeling of activated microglial markers TNFα (Supplementary Figure S3A), cyclooxygenase 2 (COX2) (Supplementay Figure S3B) as well as qPCR of TNFα (Figure 4B; *p* < 0.05), IL-6 (Figure 4C; *p* < 0.01), and COX-2 (Figure 4D, *p* < 0.05). Together, these results indicate robust negative regulatory effects of ES on microglia activation and pro-inflammatory cytokine production.

### Electric stimulation attenuates LPS-induced transcriptome profile changes in microglial cells

**FIGURE 5:**
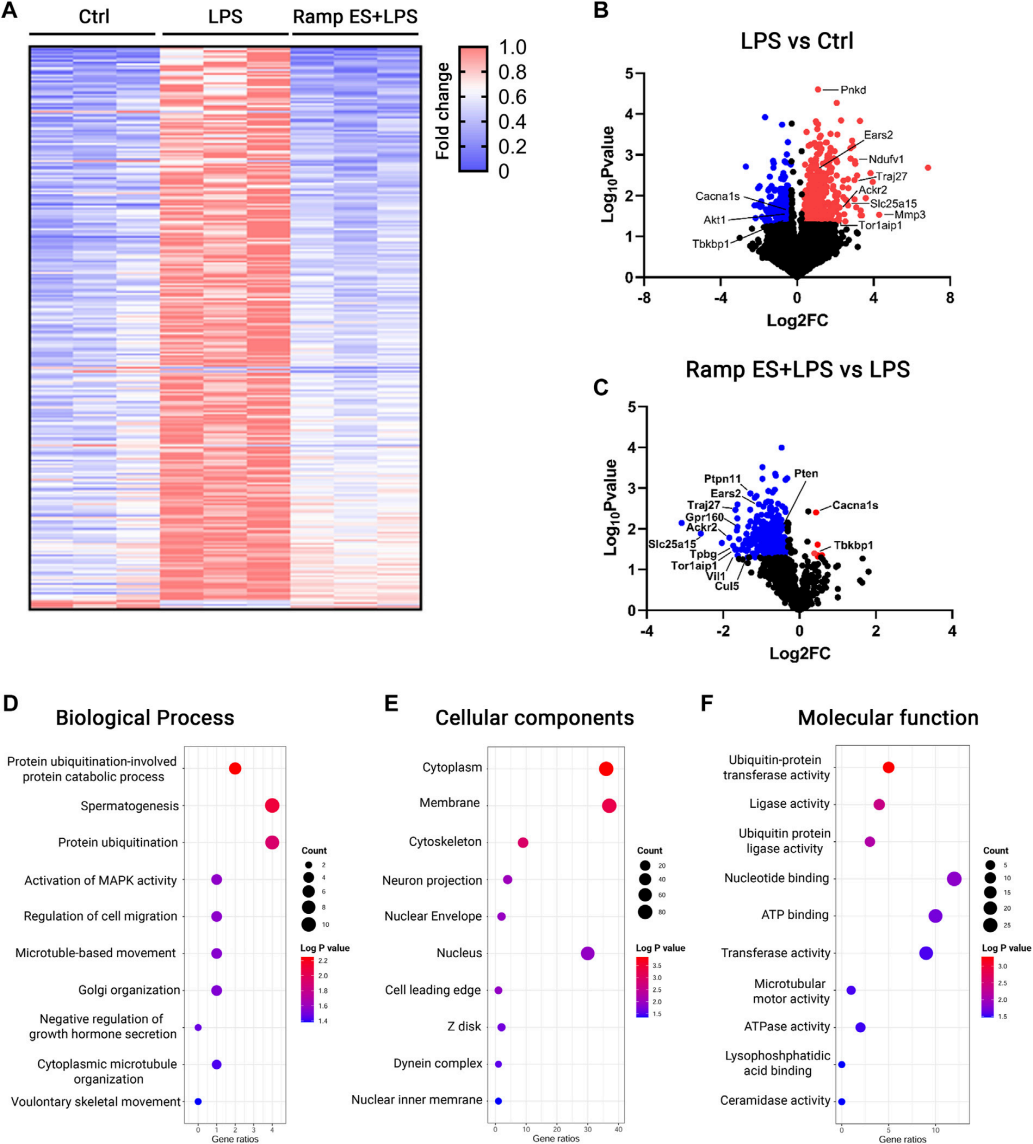
RNA-seq analysis for the transcriptome profiles of LPS-challenged BV-2 cells with or without ES-treatment. **(A)** Heatmap of DEGs in Control, LPS, and Ramp ES + LPS treated groups; the values were normalized as fold change over the control group. **(B,C)** Volcano plots of Control vs LPS **(B)** and LPS vs Ramp ES + LPS **(C)**. **(D–F)** Dot plots of top ten GO terms through analysis of ES-suppressed genes that were divided into the categories of Biological process **(D)**, Cellular components **(E)**, and Molecular function **(F)**.

To comprehensively study the effects of ES in microglia, we performed RNA sequencing. The RNA speciments were extracted from BV-2 cultures treated with ramp ES, followed by a challenge with LPS. Cultures challenged by LPS alone were served as positive controls, and those treated by PBS were used as negative controls. Following RNA-seq data normalization (Supplementary Figure S4A), we achieved a clear separation between the populations of the experimental groups in the principal component (PC) analysis (Supplementary Figure S4B). Using the cutoff of *p*-value < 0.05 and fold changes >1.2, we detected 923 differentially expressed genes (DEGs)—686 upregulated and 237 downregulated genes—induced by LPS compared to PBS-treated controls. LPS treatment induced a pro-inflammatory DEG profile, showing increases in Matrix metalloproteinase (Mmp3), energy metabolism-related genes (Slc25a15, Ears2, Ndufv1), inflammation-related genes (IL-6, TNFα, Traj27, Ackr2), and others (Figure 5B). The microglia cell ATPase activity (DYNC2H1, DYNC1H1, DNAH12, MACF1, DDX3X, ABCB1A, ABCF3, MDN1); ATP binding (CAMK2D, DDX3X, KIF11, TTBK2, GK5, SLK, ACVR1C, BTAF1, ATP2B4, and others) as well as NADP binding (DHFR, ME1, FMO2, PGD) were upregulated in LPS treated group relative to control. Furthermore, microglia motility-related (DYNC2H1, DYNC1H1, DNAH12, STARD9, KIF11) genes were upregulated (Supplementary file DEG_LPSvsCtrl).

In contrast, ES pretreatment to LPS-challenged (Supplementary file DEG_LPSvsCtrl.xls) BV-2 cultures significantly suppressed 294 out of 923 LPS-induced DEGs. These include the expression of the above-mentioned energy (Supplementary file DEG_ES_LPSvsLPS.xlsx) metabolism and motility-related genes that were upregulated by LPS. Inflammation-related genes such as (Traj27, Ackr2) were also downregulated while inducing only a few gene upregulations (Figure 5C). Only six genes were significantly upregulated in the ES pretreatment to LPS-challenged; they included L-type voltage-dependent calcium channel alpha 1S (Cacna1s), TBK Binding Protein 1 (Tbkbp1), Zfp771), and three unknown transcripts Gm38134, Gm13432, Gm28959 (Figure 5C; Supplementary Tables S1–3). The RNA-seq data were further validated by qPCR of selected genes, including Tbkbp1 (*p* = 0.047; Log2 FC = 0.481; Supplementary Figure S4A) and Atp2b4 (*p* = 0.022; Log2 FC = -0.997; Supplementary Figure S4B).

The gene ontology (GO) analysis of the ES-downregulated DEGs compared to the LPS-challenged group revealed decreases in cell migration, microtubule-based movement (Figure 5D; Supplementary Table S2), cytoskeleton (Figure 5E; Supplementary Table S3), and microtubule motor activity-related genes (Figure 5E; Supplementary Table S4); nearly all of this genes are significantly upregulated in BV-2 cells challenge with LPS when compared with control. These results align with the above observations that ES reduced microglial motility. Furthermore, ES also decreased DEGs associated with ATPase activity (Figure 5E; Supplementary Table S4), ATP binding (Figure 5E; Supplementary Table S4), and NADP (Supplementary Table S4) binding—genes underlying the control of cellular metabolism and oxygen consumption. The number of metal ion binding genes (KDM5A, NRP2, GALNT15, HHIP, FCNA, ENDOV, ME1, TRIM24, PAPOLA, ZBTB39, DDX59, MBNL1) were also significantly downregulated by ES pretreatment (Supplementary Table S4) while upregulated in LPS challenged group relative to control. Together, these data further support the prominent negative effects of ES on LPS-induced microglia motility, metabolism changes, and neuroinflammatory responses.

## Discussion

In this study, we present results supporting the direct regulatory effects of the ES on microglia in their responses to LPS-induced neuroinflammation, cytokine induction, and cellular motility and cytoskeletal remodeling. These findings underscore the electrical field’s important role in previously thought electrically “non-responsive” microglial cells and bring novel aspects to future consideration of ES therapies. Chronic microglial activation and inflammation are thought to be critically contributing factors to neurodegeneration in aging and neurodegenerative disorders. Given the safety profile of low current ES in the clinic and animal testing, these findings provide a basis for future exploration of non-invasive ES as an anti-inflammation and neuroprotective therapy.

The suppressive effects of ES on neuroinflammation were supported by observations in animal models and clinical trials. Recent studies suggest beneficial effects of ES in a rat model of stroke, in which mesencephalic electric stimulation attenuated the expression of inflammatory cytokines, IFN-γ, TNF-α, and IL-1α, in the perilesional area of stimulated rats (Schuhmann et al., 2021). Goldfarb et al. report that electroconvulsive therapy reduced microglial activation or production of pro-inflammatory cytokines in the brain of mice following intracranial LPS injection (Goldfarb et al., 2021). In the patients, a randomized, triple-blind, sham-controlled study of transcranial ES in 50 stroke subjects demonstrated significant improvements in dexterity, strength, sensitivity, anxiety, and depression (Bornheim et al., 2020). While these studies do not distinguish ES’s direct or secondary effects on the microglia, our data demonstrated for the first time the direct effects of ES on microglia.

Studies using direct current suggest the electrotaxis effects of ES on non-neuronal cells, such as muscle (Ko et al., 2018) and endothelial cells, inducing an asymmetric distribution of cytoskeletal proteins and cell migration toward the cathode *in vitro* (Cortese et al., 2014). This is the first report that the biphasic ramp current effectively regulates microglial function by reducing migration capacity, suppressing the redistribution of actin and β-tubulin and cellular micropiles. This data presents fundamental differences in the biological effect of biphasic and direct electric currents. Interestingly, different waveforms of ES showed various efficacy, with biphasic ramp producing the most prominent effects on reducing microglia motility and cytoskeleton protein reorganization. Currently, little is done to compare the biological effects among different ES waveforms. In excitable cells such as cochlear neurons, the ramp waveform is reported to require less voltage than the rectangular waveform to evoke auditory brainstem response, suggesting that the ramp waveform is more efficient in generating electrical field in tissues (Navntoft et al., 2020). Our previous reports showed that the ramp waveform is also more efficient in evoking a regenerative response of Müller cells, likely making it a preferable form of ES with optimal neuroprotective effects in the eye through mediating multiple cell types (Enayati et al., 2020; Yu et al., 2020). Furthermore, as ramp ES exerts its effect at a low current and voltage, this may also reduce the risk of complications in patients. While microcurrent ES is reported to be generally safe with minimal side effects, further investigation is needed in the future, especially the ramp waveforms, which have not been tested in a clinical setting.

**FIGURE 6:**
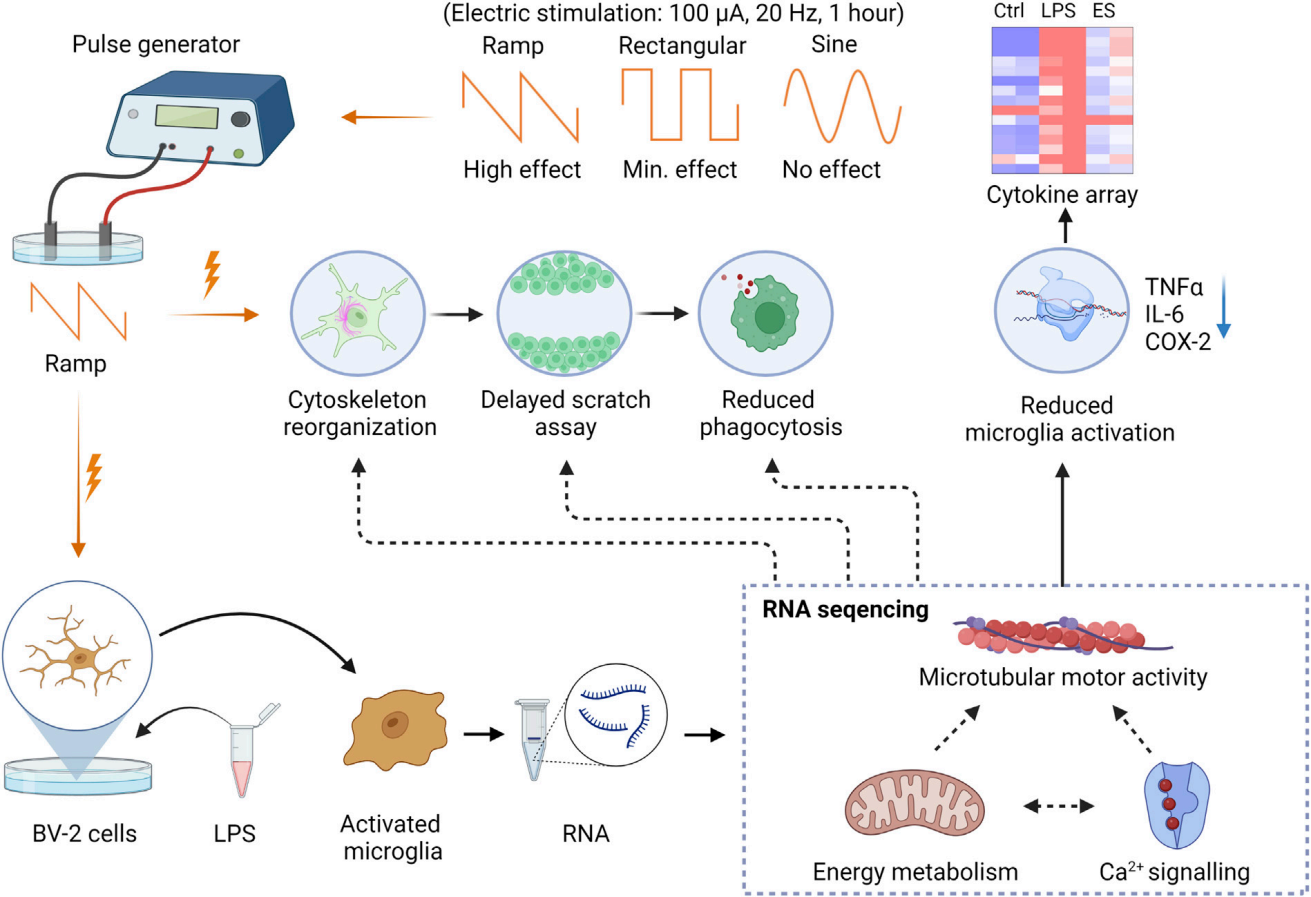
Graphical summary of the study findings. The Ramp ES affects the cytoskeletal organization, reducing cell motility in the scratch assay and phagocytosis. Rectangular and sine waveforms do not appear to have a prominent effect on cytoskeleton, motility, and phagocytosis. Ramp stimulation also reduces pro-inflammatory microglia activation and cytokine release by the BV-2 cells. RNA sequencing indicates genes’ downregulation in regulating cell migration, energy metabolism, and Ca^2+^ signaling.

The cytokine array results and RT-PCR analysis showed that ES reduced microglial activation and cytokine release in response to LPS. The levels of major pro-inflammatory factors, such as TNFα, IL-6, and COX-2, were reduced. These results are in line with the protective effects of ES reported in clinical studies of optic neuropathy (Sabel et al., 2011; Gall et al., 2016), acute ischemia (Fujikado et al., 2006; Oono et al., 2011; Naycheva et al., 2013), and retinal degenerative conditions (Shinoda et al., 2008; Chaikin et al., 2015; Sehic et al., 2016), among which the activation of microglia plays critical roles in their neuron loss. ES prevented the death of Müuller cells co-cultured with activated microglia (Zhou et al., 2012), reduced microglia density, and decreased the expression of IL-6 and COX-2 (Fu et al., 2018). Yin et al. found that ES significantly reduced TNF-α production after optic nerve crush and promoted RGCs survival by reducing microglial activation and TNF-α production (Yin et al., 2016). Our bulk RNA sequencing analysis corroborated with the functional studies and demonstrated significant downregulation of substantial numbers of genes related to microglial motility, cytoskeleton, phagocytosis, and metabolism. In agreement with our previous report on Müller cells, ES may involve Ca^2+^ signaling to mediate cellular functions. We observed decreased expression of Cacna1s, a subunit of the L-type voltage-gated Ca^2+^ channels (L-VGCC) in ES-treated BV-2 cells after the LPS challenge. Intracellular [Ca^2+^] ([Ca^2+^]_i_) is an important messenger that mediates multiple signaling pathways, including cell motility. In the microglia, an increased [Ca^2+^]_i_ levels were shown to associate with their activation *in vitro* and *in vivo*, and inhibition of L-VGCC using L-gated calcium channel blockers suppressed microglial activation (Saddala et al., 2020). In association with it, we also observed prominently upregulated TBK1, a potent anti-inflammatory mediator of microglia, in ES-treated cultures compared to those treated with LPS alone. It is reported that TBK1 inhibition resulted in increased production of IL-17 and IFNγ (Keren-Shaul et al., 2017) and microglial infiltration, which was accompanied by an increase in astrocytic scar formation in the traumatic brain injury model through activation of the canonical pro-inflammatory NFkB pathway (Rehman et al., 2021). Together, these results provided strong evidence supporting a direct modulatory effect of ES on anti-inflammatory gene expression in microglia. The graphical summary of the findings is presented in Figure 6.

An interesting observation of our transcriptome profiling studies is that ES resulted in a global suppression of gene expression in LPS-treated BV-2 cells. Indeed, our transcriptome analysis indicated that ES inhibited the upregulation of multiple metabolism-related genes, including ATP bindings and ATPase activity, induced by LPS stimulation. Lack of ATP production may explain many observed effects of ES in microglia, including decreased mobility, phagocytosis, and Ca^2+^ flux. Transitory reduction in cellular metabolism is thought to be beneficial in ischemia-reperfusion injury, as shown in animals (Wang et al., 2011; Schatz et al., 2012; Osako et al., 2013) and clinical studies (Inomata et al., 2007; Oono et al., 2011; Naycheva et al., 2013).

## Conclusion

Together, our findings suggest that the microcurrent level of ramp ES negatively regulates microglial activation, motility, and phagocytosis through inhibition of cell metabolism-related gene expression and potentially Ca^2+^ signaling, resulting in reduced cytoskeleton remodeling and pro-inflammatory cytokine productions. Further elucidation of the connections between ES-induced changes in membrane potentials and intracellular calcium signaling *in vitro* and *in vivo* may uncover key insights into the underlying mechanisms of ES-mediated functions of microglia and other cell types in the brain and retina.

## Data Availability

The datasets presented in this study can be found in online repositories. The names of the repository/repositories and accession number(s) can be found below: SRA PRJNA854281, https://www.ncbi.nlm.nih.gov/bioproject/PRJNA854281/.
