# Supplementary material for: Direct modulation of microglial function by electrical field
Source: Front Cell Dev Biol. 2022 Sep 8;10:980775. doi: 10.3389/fcell.2022.980775 (PMC9493490; doi:10.3389/fcell.2022.980775)
Supplement: Supplementary file 2 [file DataSheet1.docx]

Direct modulation of microglial function by electrical field (Supplementary materials)

## Supplementary Figures


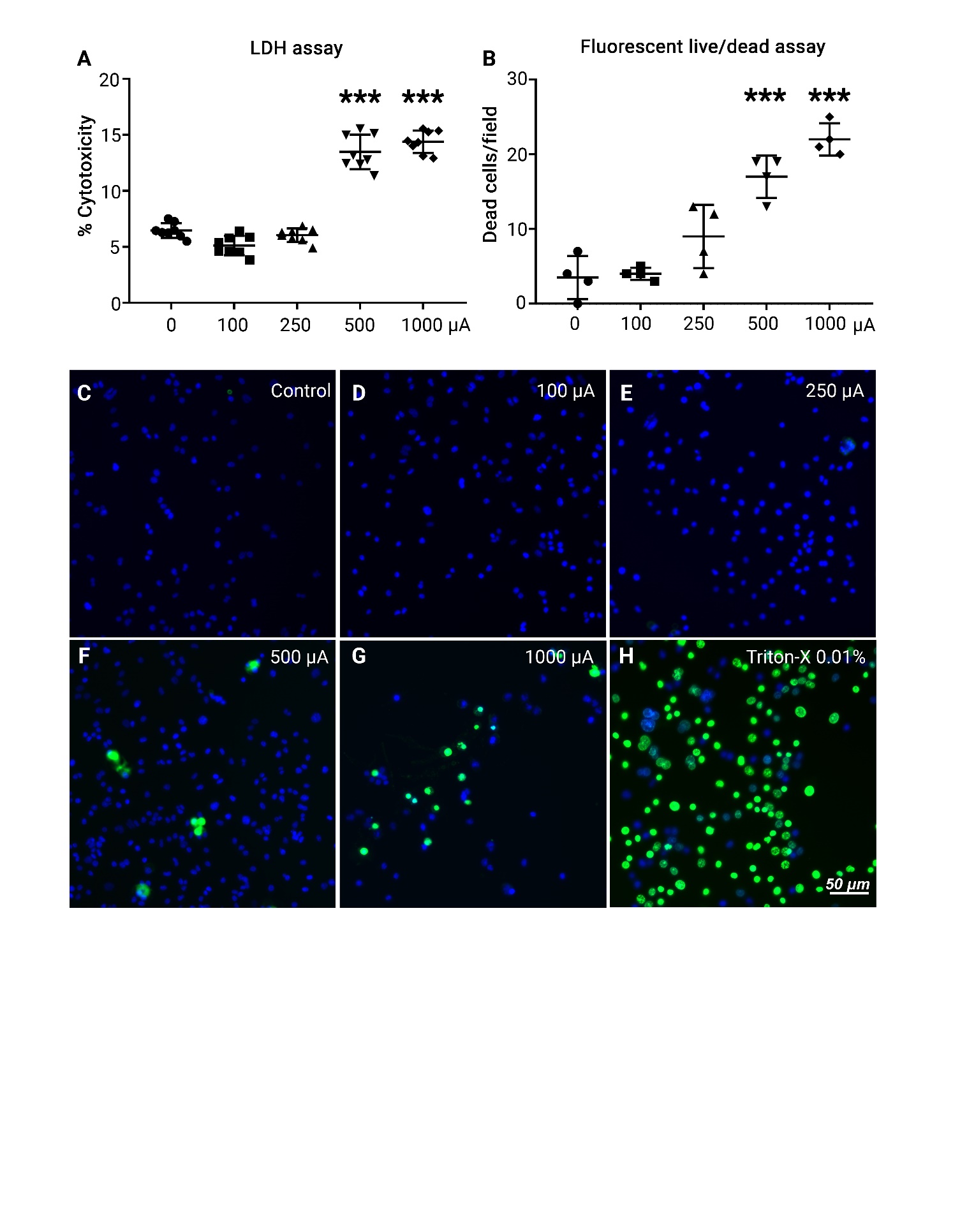


**Supplementary Figure 1. Impacts of electric stimulation on BV-2 cell survival.** (**A**) Quantification of Lactate Dehydrogenase (LDH) release assays in BV-2 cultures subjected to biphasic ramp ES of different currents (0 – 1,000 µAmp); n = 8 cultures/group. (**B-H**) Quantification (**B**) and representative images (**C-H**) of BV-2 cultures subjected to biphasic ramp ES of different currents followed by fluorescent live/dead staining assay. n = 4 cultures/group. Scale bar: 50 µm. Statistical significance was determined using one-way ANOVA with Tukey multiple comparisons. *p < 0.05; **p < 0.01; ***p < 0.001; value = means ± S.D..

**
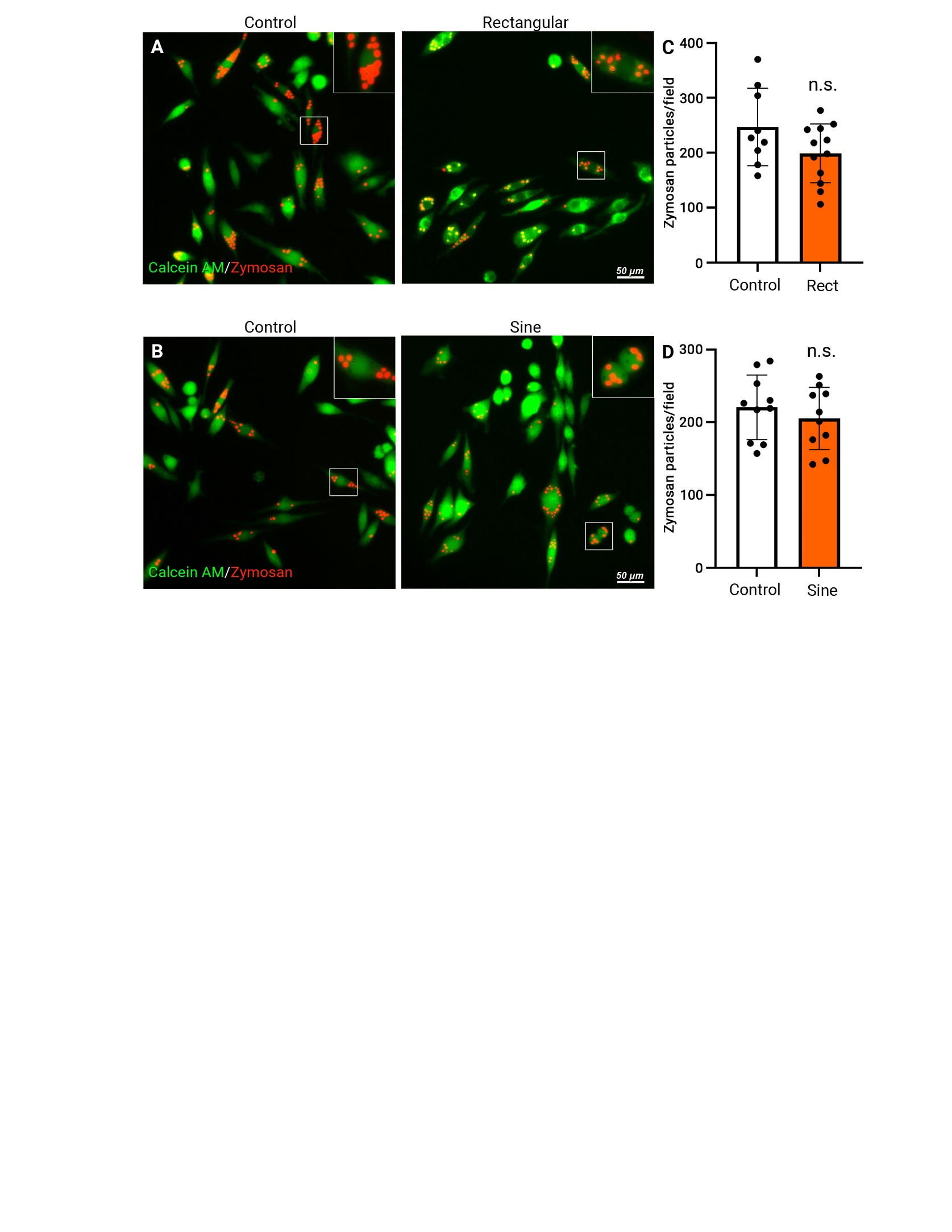
**

**Supplementary Figure 2. Ramp ES reduces phagocytosis of BV-2 cells.** (**A,B**) Representative fluorescent images of BV-2 cells cultured under a control condition or ES rectangular (A); sine (B) and incubated with fluorescent (cy3) zymosan particles (red) for 24 h and Calcein AM stain (green) was used to visualize BV-2 cells. Scale bar: 50 µm. Inserts present individual cells with zymosan particles. (**C,D**) Quantification of zymosan particles in BV-2 cells in control and rectangular ES-treated cultures (C); Control n = 10; Rectangular n = 12. Control and sine ES-treated cultures (D); Control n = 10; Sine n = 12. Statistical significance was determined by Student's t-test. n.s. p > 0.05; value = means ± S.D.

**
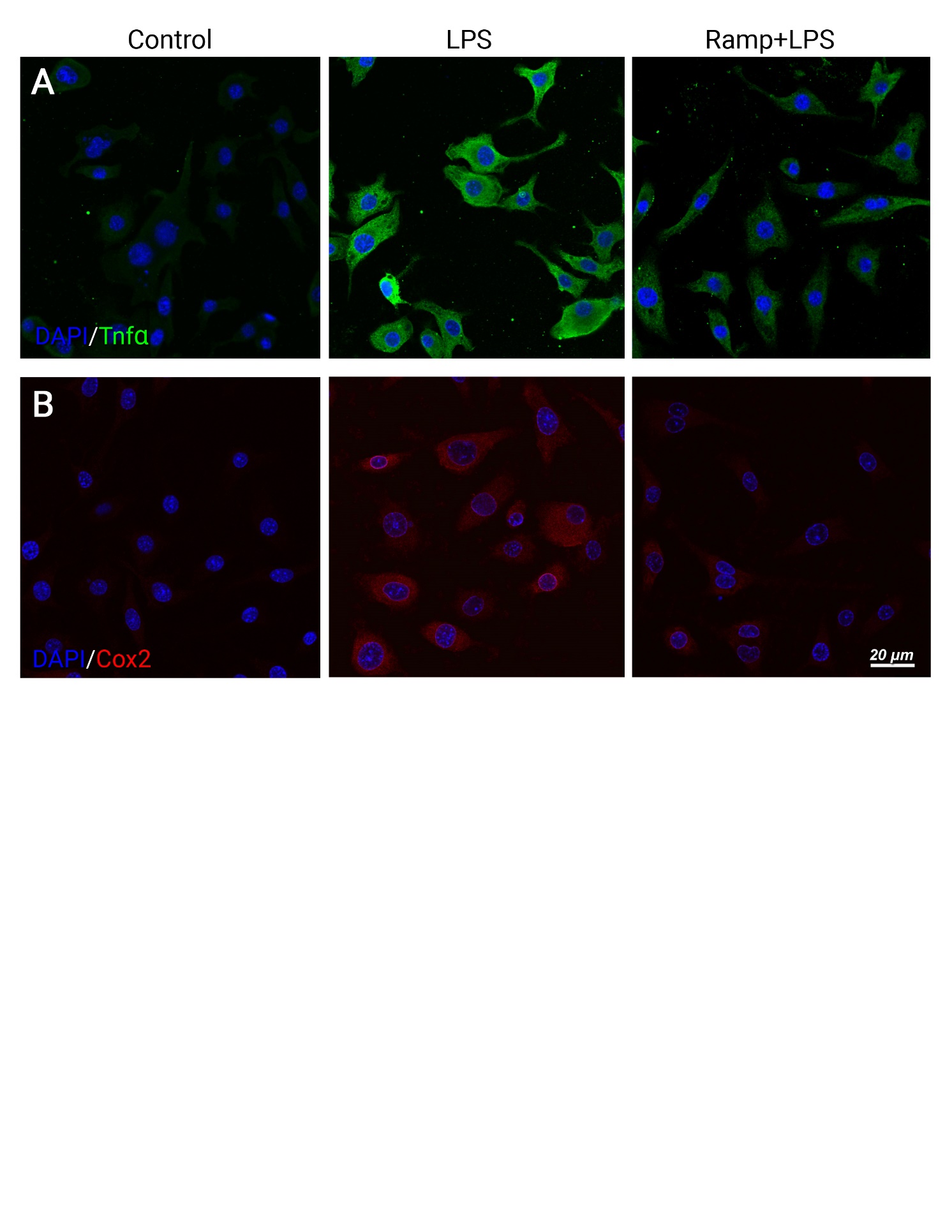
**

**Supplementary Figure 3.** Electric stimulation with ramp waveform reduces BV-2 cell activation in response to LPS stimulation. Results of immunostaining demonstrated changes in levels of of TNF-α (A); and COX-2 (B) in BV-2 cells subjected to ES biphasic ramp at 24 h after LPS stimulation. Scale bar: 20 µm.

**
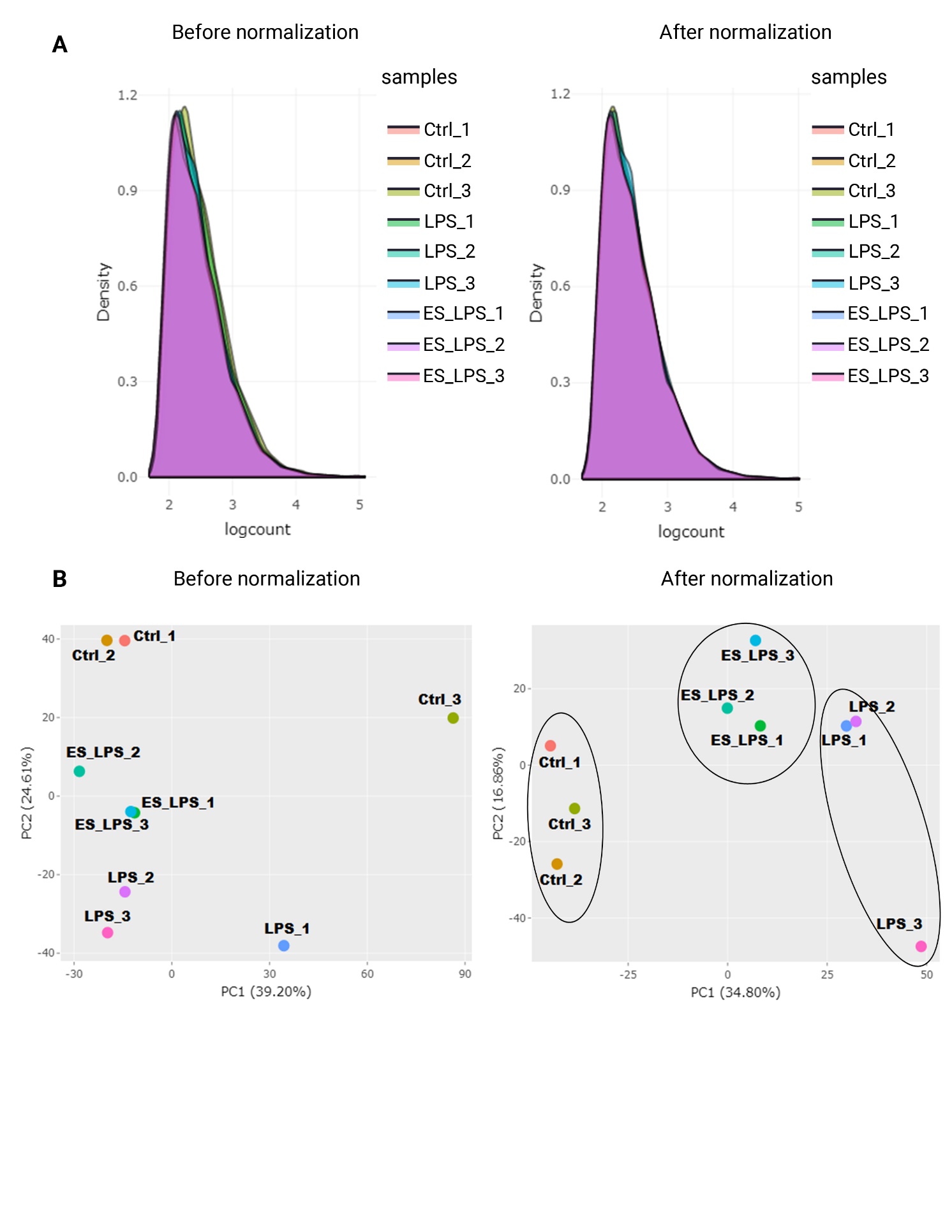
**

**Supplementary Figure 4. RNA sequencing data density plot and principal component (PC) analysis.** Density plot (A, B) and PC analysis (C,D) of the datasets before and after normalization.

**
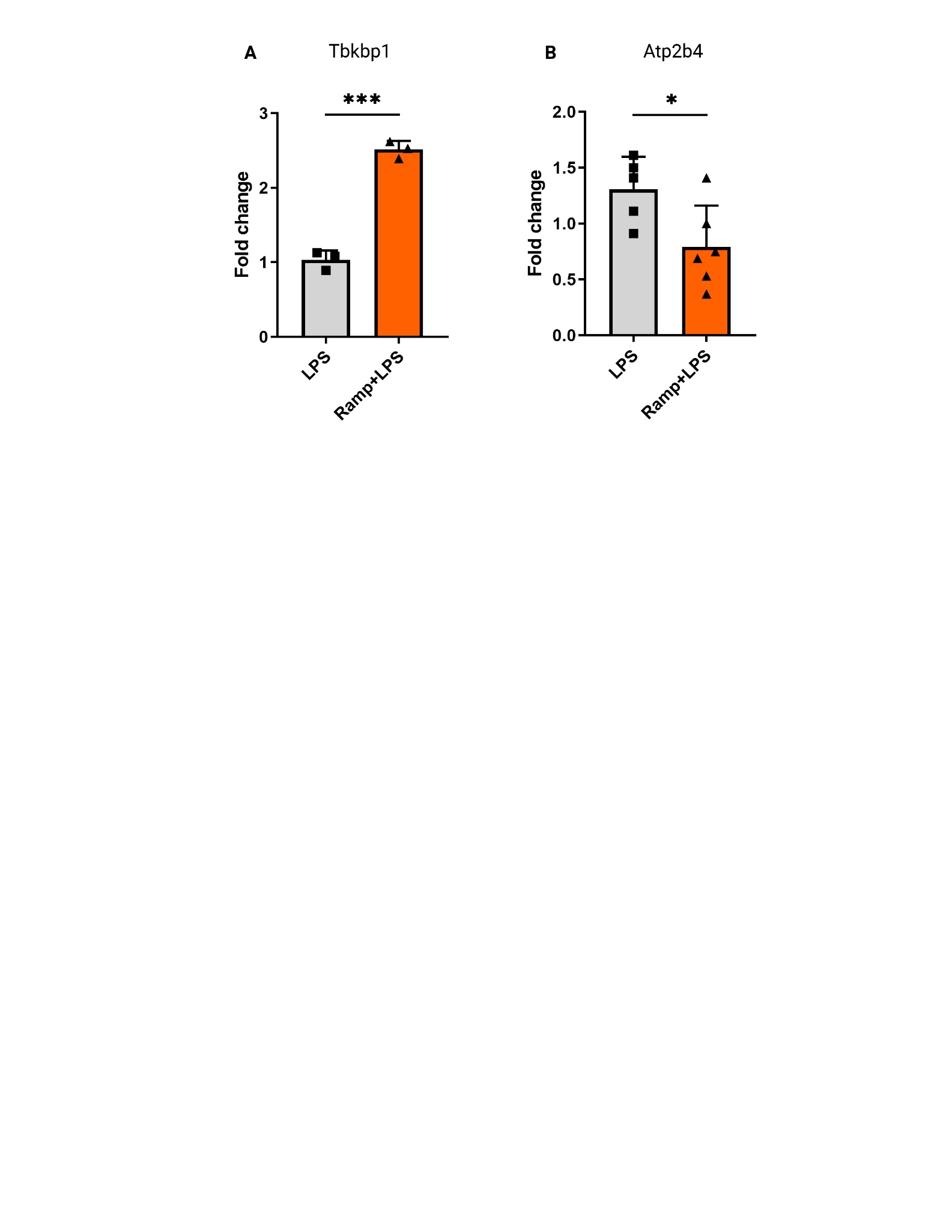
**

**Supplementary Figure 5.** Resutls of qPCR analysis of the Tbkbp1 (A) and Atp2b (B) in LPS and Ramp+LPS BV-2 cells. Statistical significance was determined using the Student T-test. *p < 0.05; ***p < 0.001; value = means ± S.D. Tbkbp1 n=3; Atp2b4 n=6.

## Supplementary Tables

**Supplementary Table 1:** RIN values of the RNA samples used for RNA sequencing analysis

| No. | Sample Name | Concentration(ng/µl) | Volume (µl) | Total amount (µg) | RIN |
| --- | --- | --- | --- | --- | --- |
| 1 | Ctr1 | 88.84 | 18.4 | 1.63473 | 8.2 |
| 2 | Ctr2 | 85.29 | 18.7 | 1.59500 | 8.7 |
| 3 | Ctr3 | 88.17 | 17.9 | 1.57832 | 8.6 |
| 4 | ES_LPS1 | 87.89 | 18.8 | 1.65242 | 9.1 |
| 5 | ES_LPS2 | 76.75 | 18.4 | 1.41211 | 8.7 |
| 6 | ES_LPS3 | 88.02 | 19 | 1.67245 | 8.8 |
| 7 | LPS1 | 85.90 | 18.5 | 1.58916 | 9.4 |
| 8 | LPS2 | 87.45 | 19 | 1.66158 | 9.6 |
| 9 | LPS3 | 106.88 | 18.7 | 1.99874 | 9.6 |

**Supplementary Table 2: Gene ontology analysis of down-regulated transcripts (Biological Process)**

| **Term** | **Genes** | **P-value** |
| --- | --- | --- |
| GO:0016567~protein ubiquitination | BARD1, DNAH12, HACE1, HECTD1, CUL5, CRBN, CUL1, TRIM24, RBBP6, SOCS7, FBXL22 | 0.005834 |
| GO:0031122~cytoplasmic microtubule organization | SLK, DST, TUBG2, TUBG1 | 0.006693 |
| GO:0048008~platelet-derived growth factor receptor signaling pathway | ITGB3, PTEN, ABL2, PTPN11 | 0.006693 |
| GO:0007018~microtubule-based movement | DYNC2H1, DYNC1H1, DNAH12, STARD9, KIF11 | 0.009885 |
| GO:0007283~spermatogenesis | KDM5A, DRC7, TBP, STRBP, PSME4, ATP2B4, UBR2, NR2C2, ZSCAN2, BIRC3, PRSS21 | 0.012697 |
| GO:0030163~protein catabolic process | TRIM24, KLK4, UBR2, UBR1 | 0.013949 |
| GO:0042787~protein ubiquitination involved in ubiquitin-dependent protein catabolic process | HACE1, HECTD1, CUL5, CUL1, UBR4, RBBP6 | 0.014821 |
| GO:0006915~apoptotic process | ASAH2, DDX3X, PTEN, CUL1, ITPR1, CFLAR, HIPK2, VIL1, SLK, ACVR1C, MADD, TAX1BP1, BIRC3 | 0.020309 |
| GO:0042981~regulation of apoptotic process | SLK, ACVR1C, MAGED1, MADD, TRIM24, CFLAR, BIRC3 | 0.020606 |
| GO:1903828~negative regulation of cellular protein localization | TRP53INP2, TTBK2 | 0.021343 |
| GO:0006511~ubiquitin-dependent protein catabolic process | CUL5, USP34, CUL1, UBR2, UBR1, TRP53INP2 | 0.025349 |
| GO:0071596~ubiquitin-dependent protein catabolic process via the N-end rule pathway | UBR2, UBR1 | 0.031844 |
| GO:0071233~cellular response to leucine | UBR2, UBR1 | 0.031844 |
| GO:0060544~regulation of necroptotic process | CFLAR, BIRC3 | 0.042233 |
| GO:0060341~regulation of cellular localization | CAMK2D, PTEN | 0.042233 |
| GO:0060125~negative regulation of growth hormone secretion | MADD, PTPN11 | 0.042233 |
| GO:0000187~activation of MAPK activity | SPAG9, MADD, PTPN11, ADRA2B | 0.048353 |
| GO:0030334~regulation of cell migration | ADGRG3, HACE1, SLK, ITGB3 | 0.04993 |

**Supplementary Table 3: Gene ontology analysis of down-regulated transcripts (Cellular Component)**

| **Term** | **Genes** | **P-value** |
| --- | --- | --- |
| GO:0005737~cytoplasm | CRTC1, HHIP, RDM1, ENDOV, SLK, SACS, TRIM24, PAPOLA, DIP2B, ARHGEF40, EARS2, DYNC2H1, PRKCI, MBNL1, COG5, HARBI1, TMOD3, TMOD2, STARD9, CD3EAP, PGD, IFIT1BL1, FBXW2, KLHL42, EML5, MADD, PSME4, MACF1, DRC7, CUL5, MAGED1, CHAT, ITPR1, UBR4, UBR2, UBR1, PDZD3, DHX33, ABL2, APOD, TRPM7, NPTX1, SOCS7, MDN1, PLK4, OSBPL9, BARD1, DNAH12, HACE1, HPS3, VIL1, BFSP2, CDK6, CNOT11, KDM5A, DDX3X, GDI1, ITGB3, PTEN, KIF11, SYNE1, ME1, TRP53INP2, DDX59, TBP, DST, STRBP, TCF12, WIPI1, TUBG2, TUBG1, MED28, LSG1, ARHGEF9, BLVRA, BIRC3, CAMK2D, FYB, STRN4, FBXL22, GK5, HECTD1, ACVR1C, CUX1, TNKS2, CLMN, ALOX5, ANKAR, RBBP6, SPAG9, DYNC1H1, CRBN, TPBG, ERAP1, PTPN11, CFLAR, HIPK2, FKBP1B, ALMS1, GPD1L, PIK3AP1, EIF3A | 1.06E-05 |
| GO:0016020~membrane | MTCH2, GALNT15, HHIP, ZDHHC2, PLB1, FCNA, TMEM89, DIP2B, SBF2, DYNC2H1, PRKCI, COG5, COX7A2L, MADD, GPR160, MACF1, TMEM87B, RPN2, MAGED1, ITPR1, UBR4, TOR1AIP2, LPAR4, CUZD1, TOR1AIP1, TMEM25, ADGRG3, PDZD3, TRPM7, SOCS7, MDN1, OSBPL9, SPECC1, HACE1, INTS2, IL31RA, GNAO1, ACER2, BFSP2, MAVS, MANSC4, DMXL2, PAM, SLC26A6, PCDHGB8, NRP2, DDX3X, ITGB3, DOCK8, KIF11, SLC7A11, SYNE1, PRSS21, TMEM44, APPL1, DST, VPS13C, ZDHHC12, TMC7, WIPI1, TMEM42, IL17RA, GNL3, MED28, SLC25A15, TMIGD1, LSG1, IGDCC4, PIGG, CAMK2D, ASAH2, DERL2, STRN4, TMEM168, ACVR1C, CUX1, TNKS2, CLMN, ALOX5, FCHO2, ANKAR, TAS1R2, STX3, ABCB1A, ABCF3, FKTN, DYNC1H1, P2RY10, CRBN, TPBG, ERAP1, ATP2B4, PTPN11, FMO2, TMCO3, PRSS32, DPY19L1, FKBP1B, ACKR2, ESYT2, GPD1L, PIK3AP1, EIF3A | 7.05E-05 |
| GO:0005856~cytoskeleton | MACF1, DRC7, UBR4, SLC7A11, KIF11, SYNE1, ABL2, RBBP6, PLK4, DYNC2H1, DNAH12, DYNC1H1, DST, STRBP, TMOD3, STARD9, TMOD2, WIPI1, TUBG2, TUBG1, KLHL42, MED28, VIL1, EML5, BFSP2, CDK6, EIF3A | 1.48E-04 |
| GO:0005634~nucleus | KDM5A, MTCH2, DDX3X, CRTC1, HHIP, ITGB3, SETD7, PTEN, AFF4, SYNE1, RDM1, ENDOV, TMEM89, TRIM24, PAPOLA, DIP2B, TRP53INP2, APPL1, NOM1, DDX59, MBNL1, PRKCI, TSPYL1, TBP, DST, STRBP, HARBI1, TCF12, STARD9, CD3EAP, PGD, FBXW2, GNL3, MED28, LSG1, MADD, PSME4, RHOX2H, BIRC3, ZFP451, CAMK2D, ZFP423, RPN2, CUL5, MAGED1, UBR4, TOR1AIP2, UBR2, NR2C2, NUGGC, TOR1AIP1, FYB, TTBK2, HECTD1, ZFP13, DHX33, CUX1, TNKS2, ZKSCAN6, ALOX5, ANKAR, RBBP6, SOCS7, RHOX3H, MDN1, FKTN, PLK4, SPECC1, BARD1, HACE1, CRBN, CPSF3, INTS2, PTPN11, HIPK2, FOXR2, PRPF39, CDK6, CMSS1, CNOT11, ASXL2, ALMS1, UBA3, TRIP11, PSMG2, NOL11, PIK3AP1, ZSCAN2, EIF3A | 3.26E-04 |
| GO:0005874~microtubule | DYNC2H1, DYNC1H1, DNAH12, MACF1, EML5, DST, STARD9, KIF11, TUBG2, TUBG1, EIF3A | 0.002798 |
| GO:0031252~cell leading edge | PRKCI, SLK, DOCK8, RAPH1, TUBG1 | 0.003009 |
| GO:0005635~nuclear envelope | TNKS2, DST, ALOX5, ITPR1, TOR1AIP2, TOR1AIP1, SYNE1 | 0.007093 |
| GO:0005865~striated muscle thin filament | TMOD3, TMOD2, NEB | 0.009494 |
| GO:0043005~neuron projection | GNAO1, GDI1, CUX1, CHAT, TMOD2, PTEN, ATP2B4, TRPM7, NPTX1, STX3, PAM | 0.015129 |
| GO:0000242~pericentriolar material | TNKS2, TUBG2, TUBG1 | 0.017228 |
| GO:0005730~nucleolus | PLK4, KDM5A, NOM1, TSPYL1, CRBN, SETD7, ITPR1, CD3EAP, GNL3, RDM1, ENDOV, DHX33, TRIM24, RBBP6, NOL11, EIF3A, MDN1 | 0.018857 |
| GO:0016605~PML body | RDM1, PTEN, TRP53INP2, ZFP451, HIPK2 | 0.019595 |
| GO:0005814~centriole | PLK4, ALMS1, STARD9, TUBG1, TTBK2 | 0.031272 |
| GO:0045177~apical part of cell | DYNC2H1, PRKCI, PDZD3, TUBG1, ABCB1A | 0.034947 |
| GO:0030286~dynein complex | DYNC2H1, DYNC1H1, DNAH12 | 0.043104 |
| GO:0030018~Z disc | DST, FKBP1B, ATP2B4, NEB, FBXL22 | 0.044098 |

**Supplementary Table 4: Gene ontology analysis of down-regulated transcripts (Molecular Function)**

| **Term** | **Genes** | **P value** |
| --- | --- | --- |
| GO:0004842~ubiquitin-protein transferase activity | BARD1, ZYG11B, HACE1, CUL1, UBR4, UBR2, UBR1, KLHL42, HECTD1, HERC1, TRIM24, RBBP6, BIRC3 | 2.47E-04 |
| GO:0016874~ligase activity | BARD1, HACE1, UBR4, UBR2, UBR1, HECTD1, HERC1, UBA3, TRIM24, RBBP6, EARS2, ZFP451, BIRC3 | 6.30E-04 |
| GO:0005515~protein binding | KDM5A, NRP2, HHIP, ITGB3, SETD7, PTEN, RAPH1, AFF4, SYNE1, FCNA, SLK, ME1, TRIM24, PRKCI, TBP, DST, TCF12, CD3EAP, TUBG1, FBXW2, GNL3, MED28, TAX1BP1, BIRC3, MACF1, CAMK2D, ZFP423, CUL5, MAGED1, ITPR1, CUL1, TOR1AIP2, UBR2, UBR1, NR2C2, PHYHIP, TOR1AIP1, FYB, PDZD3, HECTD1, ALOX5, ABL2, RICTOR, TAS1R2, STX3, ABCF3, SOCS7, PLK4, SPAG9, PTPN11, NEB, HIPK2, VIL1, GNAO1, BFSP2, MAVS, CDK6, FKBP1B, UBA3, TRIP11, ACKR2, PIK3AP1, SLC26A6, EIF3A | 0.001427 |
| GO:0016887~ATPase activity | DYNC2H1, DYNC1H1, DNAH12, MACF1, DDX3X, STARD9, ABCB1A, ABCF3, MDN1 | 0.001593 |
| GO:0061630~ubiquitin protein ligase activity | HECTD1, CUL5, CUL1, TRIM24, UBR2, RBBP6, UBR1, FBXL22 | 0.006221 |
| GO:0005524~ATP binding | CAMK2D, DDX3X, KIF11, TTBK2, GK5, SLK, ACVR1C, BTAF1, DHX33, PAPOLA, ABL2, TRPM7, ABCB1A, ABCF3, EARS2, MDN1, PLK4, DYNC2H1, DNAH12, DYNC1H1, PRKCI, DDX59, PKLR, STARD9, ATP2B4, HIPK2, CDK6, UBA3 | 0.006974 |
| GO:0050661~NADP binding | DHFR, ME1, FMO2, PGD | 0.008713 |
| GO:0003777~microtubule motor activity | DYNC2H1, DYNC1H1, DNAH12, STARD9, KIF11 | 0.009507 |
| GO:0000166~nucleotide binding | CAMK2D, DDX3X, KIF11, NUGGC, GK5, RDM1, SLK, ACVR1C, DHX33, PAPOLA, ABL2, TRPM7, ABCB1A, ABCF3, EARS2, PLK4, DYNC2H1, DNAH12, DYNC1H1, PRKCI, DDX59, PKLR, STARD9, ATP2B4, TUBG2, TUBG1, HIPK2, GNL3, GNAO1, LSG1, CDK6, UBA3, BLVRA | 0.010644 |
| GO:0046872~metal ion binding | KDM5A, NRP2, GALNT15, HHIP, FCNA, ENDOV, ME1, TRIM24, PAPOLA, ZBTB39, DDX59, MBNL1, PRKCI, DST, HARBI1, TAX1BP1, BLVRA, BIRC3, ZFP451, MACF1, ZFP423, UBR4, KLK4, UBR2, UBR1, NR2C2, HECTD1, ACVR1C, ZFP13, TNKS2, ZKSCAN6, ALOX5, ABL2, TRPM7, RBBP6, NPTX1, BARD1, CRBN, PKLR, CPSF3, ERAP1, ATP2B4, VIL1, GNAO1, ASXL2, ZFP790, ESYT2, PAM, ZSCAN2 | 0.023909 |
| GO:0016740~transferase activity | CAMK2D, RPN2, GALNT15, SETD7, CHAT, ZDHHC2, GK5, SLK, HECTD1, ACVR1C, TNKS2, PAPOLA, ABL2, TRPM7, FKTN, PLK4, PRKCI, HACE1, PKLR, ZDHHC12, HIPK2, DPY19L1, CDK6, EOGT, BIRC3 | 0.030174 |
| GO:0003779~actin binding | VIL1, MACF1, DST, CLMN, TMOD3, TMOD2, TRPM7, MED28, SYNE1 | 0.032088 |
| GO:0017040~ceramidase activity | ACER2, ASAH2 | 0.04286 |
| GO:0035727~lysophosphatidic acid binding | VIL1, LPAR4 | 0.04286 |
| GO:0017112~Rab guanyl-nucleotide exchange factor activity | DENND2C, MADD, SBF2 | 0.044627 |
